# A large nationwide population-based case–control study of the association between intussusception and later celiac disease

**DOI:** 10.1186/1471-230X-13-89

**Published:** 2013-05-16

**Authors:** Jonas F Ludvigsson, Agneta Nordenskjöld, Joseph A Murray, Ola Olén

**Affiliations:** 1 Clinical Epidemiology Unit, Department of Medicine, Karolinska University Hospital and Karolinska Institutet, Karolinska, Sweden; 2 Department of Pediatrics, Örebro University Hospital, Örebro, Sweden; 3 Division of Gastroenterology and Hepatology, Departments of Medicine and Immunology, Mayo Clinic College of Medicine, Rochester, NY, USA; 4 Department of Women’s and Children’s Health, Karolinska Institutet and Department of Pediatric Surgery, Karolinska University Hospital, Karolinska, Sweden; 5 Sachs’ Children’s Hospital, Stockholm South General Hospital, Stockholm, Sweden

**Keywords:** Celiac, Coeliac, Gluten, Inflammation, Intussusception, Population-based

## Abstract

**Background:**

Case reports and case series studies suggest a positive association between intussusception and celiac disease (CD).

**Methods:**

We contacted Sweden’s 28 pathology departments and obtained data on 29,096 patients with biopsy-verified CD (equal to Marsh stage 3) through biopsy reports. Patients with CD were matched for age, sex, calendar period and county of residence with up to five reference individuals from the general population (n = 144,522). Cases of intussusception were identified from nationwide inpatient, hospital-based outpatient and day-surgery data from the Swedish Patient Register.

Odds ratios (ORs) for future CD in patients with intussusception were estimated using conditional logistic regression.

**Results:**

34 (0.12%) individuals with CD had a diagnosis of intussusception vs. 143 (0.10%) reference individuals, suggesting that intussusception was not a risk factor for later CD (OR = 1.17; 95% confidence interval (CI) = 0.82–1.67). The OR for CD in patients with at least two records of intussusception was 0.40 (95% CI = 0.06–2.99).

In contrast, a post-hoc analysis showed that CD was associated with a statistically significantly increased risk of intussusception after CD diagnosis (hazard ratio = 1.95; 95% CI = 1.01–3.77); however, this analysis was based on only 12 cases with both CD and intussusception.

**Conclusion:**

We found no association between intussusception and future CD; and a mostly modest increased risk of intussusception after a diagnosis of CD.

## Background

Intussusception is a condition in which a segment of intestine invaginates into another section of intestine. Signs and symptoms of intussusception include acute pain, nausea, lethargy, vomiting and sometimes bleeding from the rectum (“red currant jelly”) [1]. Although intussusception generally remits spontaneously, in a number of cases either contrast enema or surgery is needed to treat the disorder [2]. The causes of intussusception include infections and vaccination with rotavirus vaccine, but organic disorders such as malignancy tend to predominate in adults.

Celiac disease (CD) is a chronic inflammatory small intestinal disorder that occurs in about 1–2% of the general population [3,4]. Undiagnosed CD is characterized by small bowel inflammation [5] and will sometimes cause small bowel wall edema [6], intestinal lymph node swelling [7] and dysmotility [7] but also ulcers and strictures [8]. It has therefore been suggested that untreated CD may be linked to intussusception; a number of case-reports and case series substantiate this hypothesishesis [9-14]. In a recent paper from a tertiary institution in the US [14], 3/252 (1.2%) of children with newly diagnosed CD had a history of intussusception compared with 0.07% of the child population attending this center. This suggests intussusception may be associated with CD, however, due to small numbers or lack of controls, none of these studies have estimated relative risks or odds ratios (ORs) [9-14].

The main objective of this study was therefore to examine the association between intussusception and later biopsy-verified CD in a large nationwide population-based case–control study. In a post-hoc analysis we examined the risk of intussusception in patients who already had a diagnosis of CD.

## Methods

We identified patients with intussusception through the Swedish Patient Register [15] (data on inpatient care, hospital-based outpatient care and day-care surgery). Intussusception data were linked to data on CD obtained from biopsy reports at Sweden’s 28 pathology registers [16]. Linkages were performed using the Swedish personal identity number (PIN) [17].

### Intussusception

Cases with intussusception were identified from the Swedish Patient Register [15] by reference to relevant International Classification of Disease (ICD) codes ICD-7: 570.0; ICD-8: 560.0; ICD-9: 560A; and ICD-10: K56.1. In subanalyses we restricted intussusception to cases with surgery or to those who had radiological intervention (code TJG30 or 4780) for intussusception. In a separate analysis we examined the risk of CD in patients with at least two recorded health care contacts that were due to intussusception.

The Swedish Patient Register started in 1964 and since 1987 the register has nationwide coverage. Day-surgery data were added from 1997 and hospital-based outpatient data since 2001 [15].

### Celiac disease

In 2006–2008 we collected small intestinal biopsy report data from all pathology departments (n = 28) in Sweden. The biopsies were performed between 1969 and 2008. IT personnel carried out computerized searches for arrival date of biopsies, PIN [17], morphology and topography (duodenum and jejunum). CD was defined as having a biopsy with villous atrophy (VA, equals Marsh stage 3) [18] according to the Swedish SnoMed classification. We did not require patients to have a positive antibodies against tissue transglutaminase (TTG), endomysium (EMA), or gliadin for a CD diagnosis, but in a random subset of patients with VA and available data on CD serology about 88% were positive for either of these antibodies at the time of biopsy [16]. An earlier validation of 114 patients with VA found that 108 (95%) had CD [16]. Approximately 79% of individuals with CD had gastrointestinal symptoms before biopsy and 35% had anemia.

### Controls

Each individual undergoing biopsy was matched with up to five controls for age, sex, calendar period and county of residence. Controls were identified from the Total Population Register by the Swedish government agency, Statistics Sweden and had no previous duodenal/jejunal biopsy.

We then excluded individuals whose biopsy may have originated from the ileum, CD individuals lacking a serial number from Statistics Sweden or having no matched controls since all analyses were carried out per stratum. The remaining individuals were identical to those in our study on mortality in CD [19]. Thus, the final sample on which this study is based was 29,096 individuals with CD and 144,522 matched controls.

### Statistics

We used conditional logistic regression to estimate ORs for CD and earlier intussusception. The conditional approach entails that each individual with CD is only compared with his or her controls within the same stratum. We also present percentages of CD patients and controls with a previous diagnosis of intussusception.

In pre-defined subanalyses we examined intussusception and CD in relation to sex, age (0–19, 20–39, 40–59 and ≥60 years at age of diagnosis) and calendar period (1989, 1990–1999 and 2000-) at CD diagnosis.

In another pre-defined subanalysis we examined the association between intussusception and CD in children aged <2 years (CD: n = 4,589). We did so for two reasons. First, in Sweden, CD is often diagnosed in this age group [20], and second, as opposed to adults in which intussusception may be caused by cancers [11], underlying cancers are unusual in childhood when most intussusception is idiopathic. We therefore hypothesized that the association with CD would be strongest with idiopathic intussusception in infancy and childhood. For consistency, we also examined the risk of intussusception in individuals diagnosed with CD ≥2 years of age (CD: n = 24,507). In a post-hoc analysis we excluded all intussusception occurring before the age of 2 years (not equal to the previous analysis in which we examined risk of CD after the age of 2 years but included intussusceptions occurring before age 2) and calculated OR for future CD.

We also examined the risk of future CD in patients with at least 2 health care contacts for intussusception (repeated intussusceptions) of which at least one intussusception had to occur before CD diagnosis. Having at least 2 records of intussusception will increase the likelihood that the patient really had intussusception.

Cancer is sometimes the underlying cause of intussusception [11] and CD has been linked to both lymphoproliferative [21] and gastrointestinal cancer [22] (at least about the time of CD diagnosis [23]). To rule out that a positive association between CD and intussusception would be due to cancer we performed a separate analysis in which we excluded all individuals (CD patients and controls) who ever had a diagnosis of cancer according to the Swedish Cancer Registry (CD: n = 25,869). The Swedish Cancer Registry began in 1958. About 99% of all cancers are morphologically verified [24] and almost 100% of all cancers are reported to this register each year [24].

### Post-hoc analysis: CD and risk of future intussusception

In a post-hoc analysis we examined the risk of future intussusception in patients with CD. This analysis was done to explore whether the null relationship that we found between intussusception and later CD was independent of temporal sequence. In the prospective analysis we used a cohort study design. Individuals with CD were compared with matched reference individuals and followed from biopsy (or matching date) until first event of intussusception, emigration, death or end of follow-up (Dec 31, 2009), whichever occurred first. We used Cox regression models to calculate hazard ratios (HRs) for the risk of future intussusception in CD. All analyses were internally stratified, i.e. one individual with CD was only compared with his or her matched reference individuals (within a stratum) before a summary estimate for the whole CD population was calculated. This approach eliminates the influence of matching variables, such as sex, age, county of residence and calendar year at CD diagnosis. The prospective cohort analysis was based on 29,060 individuals with no earlier record of intussusception and 144,304 matched reference individuals.

SPSS version 20.0 (SPSS Inc, Chicago, IL, USA) was used for all analyses. Statistical significance was defined as 95% confidence intervals (CIs) for risk estimates (ORs and HRs) not including 1.0.

### Ethics

The study was approved by the regional ethical review board in Stockholm, Sweden. Since none of the participants was contacted and individual information was “anonymized” before the analyses, informed consent was not required by the board.

## Results

The median age at CD diagnosis was 30 years (range 0–95) (Table 1). Most patients with CD were diagnosed after 1990 since this study was based on computerized biopsy reports and computerized registers were usually introduced in this decade. Data on the sub-cohort of children diagnosed with CD <2 years of age are presented in Additional file 1. Slightly more than 6 of 10 study participants (all ages) were women.

**Table 1:** Characteristics of study participants

	**Matched controls**	**Patients with celiac disease**
Total, n	144,522	29,096
Age at celiac diagnosis, years (median, range)	*	30; 0–95
Age 0–19, n (%)	58,852 (40.7)	11,802 (40.6)
Age 20–39, n (%)	26,385 (18.3)	5,312 (18.3)
Age 40–59, n (%)	32,254 (22.3)	6,477 (22.3)
Age ≥60, n (%)	27,031 (18.7)	5,505 (18.9)
Entry year (median, range)	1998, 1969–2008	1998, 1969–2008
Females, n (%)	89,544 (62.0)	18,005 (61.9)
Males, n (%)	54,978 (38.0)	11,091 (38.1)
*Calendar year*		
−1989, n (%)	20,378 (14.1)	4,105 (14.1)
1990–99, n (%)	59,874 (41.4)	12,059 (41.4)
2000, n (%)	64,270 (44.5)	12,932 (44.4)
*Country of birth*		
Nordic#	136,279 (94.3)	28,139 (96.7)
*Data on intussusception*		
Intussusception n (%)	143 (0.10)	34 (0.12)
Age at first intussusception, years (median, range)	1 (0–60)	3 (0–68)
Intussusception with surgery/radiology, n (%)	31 (0.02)	8 (0.03)

* Reference individuals were matched for age. The median age at matching was 30 years (range 0–95 years).

# Sweden, Denmark, Finland, Norway and Iceland.

### Intussusception and risk of CD

Of 29,096 individuals with CD, 34 (0.12%) had a diagnosis of intussusception vs. 143/144,522 of the controls (0.10%). Hence, we found no association between intussusception and later CD (OR = 1.17; 95% CI = 0.82–1.67). The OR for having a diagnosis of CD was 1.31 (95% CI = 0.64–2.68) within 1 year after intussusception, 0.56 (95% CI = 0.17–1.78) 1–< 5 years after intussusception and 1.31 (95% CI = 0.84–2.05) ≥5 years after intussusception.

The risk of future CD was similar in females (OR = 0.97; 95% CI = 0.57–1.66) and males (OR = 1.40; 95% CI = 0.86–2.28) (Table 2), and there were no significant differences in ORs according to age at CD diagnosis or calendar year (Table 2). Adjustment for education and country of birth (Nordic vs. Non-Nordic) did not affect our ORs (data not shown).

**Table 2:** Intussusception and risk of later celiac disease

**Subgroup**	**Intussusceptions, N**		**OR; 95% CI**	**P-value**	**P for interaction**
	**Celiac disease**	**Controls**			
***Sex***					
Males	19	66	1.40; 0.86–2.28	0.176	0.323
Females	15	77	0.97; 0.57–1.66	0.922	
Age*					
<20 yrs	20	103	0.97; 0.61–1.54	0.902	0.256
20–39 yrs	8	23	1.67; 0.78–3.66	0.186	
40–59 yrs	3	6	2.34; 0.64–8.62	0.201	
60+ yrs	3	11	1.34; 0.39–4.66	0.645	
Calendar period*					
−1989	7	21	1.59; 0.71–3.56	0.258	0.292
1990–1999	14	54	1.27; 0.72–2.22	0.407	
2000–2008	13	68	0.96; 0.54–1.70	0.881	

*At time of celiac disease diagnosis.

### Subanalyses

When we restricted our dataset to children <2 years of age (at diagnosis of CD or at date of matching), 6 individuals with CD had a previous intussusception vs. 32 controls. Intussusception was no risk factor for CD in this subset of young children (OR = 0.94; 95% CI = 0.42–2.14) (Table 3). Table 3 shows additional data.

**Table 3:** Subanalyses: Intussusception and risk of later celiac disease

**Subgroup**	**Celiac disease <2 years**	**Intussusception with radiology#/surgery**
	**OR; 95% CI**	**OR; 95% CI**
***Overall***	0.94; 0.42–2.14	1.27; 0.60–2.69
***Sex***		
Males	1.55; 0.56–4.31	1.07; 0.31–3.63
Females	0.53; 0.13–2.16	1.42; 0.55–3.67
Age*		
<20 yrs	*Not estimated*	0.80; 0.24–2.62
20–39 yrs	*Not estimated*	2.88; 0.93–8.91
40–59 yrs	*Not estimated*	2.50; 0.23–27.57
≥60 yrs	*Not estimated*	*Not estimated*§
Calendar period*		
−1989	1.41; 0.44–4.54	2.31; 0.48–11.30
1990–1999	0.59; 0.14–2.39	0.94; 0.28–3.14
2000–2008	1.23; 0.15–9.82	1.33; 0.39–4.52

*At time of celiac disease diagnosis. #Radiological intervention for intussusception.

§ No cases of previous intussusception in the CD group vs. four cases in the control group.

Similarly, intussusception was no risk factor for having a diagnosis of CD after 2 years of age (OR = 1.24; 95% CI = 0.83–1.86). However, we did find a statistically significant association (OR = 2.11; 95% CI = 1.26–3.53) in a post-hoc analysis that only examined intussusceptions occurring after the age of 2 years and risk of future CD.

Restricting our exposure to intussusception with either radiological intervention or surgery, the OR for future CD was 1.27 (95% CI = 0.60–2.69).

One patient with CD vs. 13 controls had ≥2 records of intussusception (out of which at least one intussusception occurred before CD diagnosis and study entry). This outcome corresponded to a non-significant OR of 0.40 for future CD (95% CI = 0.06–2.99). In eleven of the fourteen (1 + 13) patients with ≥2 records of intussusception, did the two records correspond to the same intussusception (e.g. follow-up visit shortly after first diagnosis). All three patients with ≥30 days between the two intussusceptions (our definition of different episodes), were controls.

Excluding study participants who had a diagnosis of cancer at some stage in life, the OR for future CD in patients with intussusception was 1.10 (95% CI = 0.76–1.59) (31/25,869 CD patients vs. 140/130,041 controls had an earlier diagnosis of intussusception).

### Celiac disease and risk of future intussusception

Using a prospective cohort approach, a post-hoc analysis found that 12 of 29,060 individuals with CD had a diagnosis of intussusception after CD onset (expected n = 6), corresponding to a hazard ratio and relative risk of 1.95 (95% CI = 1.01–3.77, p = 0.046).

## Discussion

To our knowledge this is the first case–control study examining intussusception and risk of future CD. It found no association between intussusception and CD (overall OR = 1.17). In a post-hoc analysis intended to confirm the null relationship between prior intussusception and CD before diagnosis we instead found that patients with diagnosed CD were at an almost twofold increased risk of *later* intussusception.

Most literature on CD and intussusception has been limited to case reports or case series [9-13,25]. Germann *et al.* suggest that intussusception in CD has a mild clinical course [25]. Intussusception may take place in the duodenum or jejunum but may occur in other parts of the intestine. In our study we were unable to differentiate between intussusceptions in the small intestine and the colon. If intussusception is associated with CD, it is most likely associated with small intestinal intussusception and thus the inclusion of colonic intussusception may have diluted a positive relationship. Still, the overall OR for future CD was very close to 1.0, and if there had been a significant association between previous intussusception and CD, this would have appeared in our data. Reilly *et al.* reported that 1.2% of their celiac children had experienced a known intussusception [14] but since the authors do not present any statistical comparison with the general population this may or may not represent an increase. Our study differs from that of Reilly *et al.*[14] by different source populations (nationwide approach vs. tertiary institution), and larger number of celiac patients (29,096 vs. 254).

In a recent paper we studied the role of surgery in adult intussusception [11]. In that case series 8/196 (4%) patients with intussusception had CD [11]. In a paper looking at the risk of intussusception in patients with a diagnosis of CD 14/880 (1.6%) developed intussusception during follow-up [13]. In both these series, with strong associations between CD and intussusception, participants were recruited from single tertiary centers, making it is possible that this procedure selected cases with more severe CD and intussusception [11,13], or that these patients were at higher risk of undergoing investigation or contacting health care for a number of disorders. We have previously shown that the relative risk of another disorder in CD (tuberculosis) is twice as high in CD patients identified through hospital records [26] as in patients identified through biopsy reports [27]. Further, the risk of mortality in CD seems higher in patients with a hospital record of CD [28] than in patients diagnosed through biopsy reports [19]. More than 96% of pediatricians and gastroenterologists in Sweden perform a biopsy in at least 90% of patients with suspected CD before diagnosis [29]. Other strengths of our paper include the high specificity of VA for CD. When two independent reviewers manually scrutinized more than 1500 biopsy reports, very few individuals had other comorbidities than CD (0.3% of patients with VA had inflammatory bowel disease and 0.2% had Helicobacter pylori). The nationwide ascertainment of CD yielded a large number of CD cases, which contributed to high statistical power. We were therefore able to stratify for age, sex and calendar period at CD diagnosis.

Restricting our analyses to intussusception with radiological intervention and surgery did not affect the OR (1.27). Only after we restricted our analyses to individuals with intussusception after the age of 2 years, did we find a positive association between intussusception and future CD. We urge caution when interpreting these data since they made up a post-hoc finding and could be due to chance. Still, these data may reflect a true association between inflammation from undiagnosed CD in older children and adults and secondary intussusception.

This study has some limitations. We used a case–control design to examine the association between previous intussusception and future CD. This design means that we did not screen individuals with intussusception for serological markers. Still, if any, patients with intussusception would have been more likely to undergo investigation for CD and this would have driven up the OR for CD. The lack of serological data can therefore not explain our null findings for later CD risk.

We did not have access to radiological data such as computed tomography and thus could not confirm the intussusception diagnosis. However, in several analyses we increased the specificity of intussusception through various restrictions of the dataset and this had only marginal effects on the ORs. Nor did we screen CD patients for intussusception with MRI, CT or ultrasound [7]. Thus, we have no information on the association between CD and intussusception that did not require health care.

We know of no earlier incidence study of intussusception in Sweden but a study from nearby Germany found an incidence of 60/100,000 person-years in children <1 year of age [30]. When we examined control children who were born in 1987 or later (when the Swedish Patient Registry was complete) in our dataset, there were 37 intussusceptions in the first year of life corresponding to an incidence of 83/100,000 (37/44,759 person-years). This incidence suggests that the low OR of our study is unlikely to be due to underreporting of intussusceptions.

Finally, despite the large number of patients, we cannot rule out a weak association between intussusception and later CD since the upper 95% CI reached 1.67.

Early versions of Rotavirus immunizations have been linked to intussusception [31], and we lacked immunization data. However a recent study from Sweden [32] found no association between childhood immunizations and CD, and rotavirus immunizations have not yet been included in the general childhood immunization program in Sweden. Hence, biased immunization coverage in CD children is unlikely to explain our results.

## Conclusion

In conclusion, this study found no association between intussusception and CD before CD diagnosis (undiagnosed CD), but did find a twofold increased risk of intussusception after CD diagnosis. However, because only 12/29,060 (0.04%) individuals with CD developed intussusception during follow-up, intussusception is probably a rare complication in CD. This study does not support CD screening in patients with intussusception.

## Abbreviations

CD: Celiac disease; CI: Confidence interval; OR: Odds ratio; VA: Villous atrophy.

## Competing interests

The authors declared that they have no competing interest.

## Authors’ contributions

ICMJE criteria for authorship read and met: JFL, AN, JAM, OO. Agree with the manuscript’s results and conclusions: JFL, AN, JAM, OO. Designed the experiments/the study: JFL and OS. Collected data: JFL. Analyzed the data: JFL. Wrote the first draft of the paper: JFL. Contributed to the writing of the paper: AN, JAM, OO. Contributed to the study design and interpretation of the data analyses: JFL, AN, JAM, OO. Approved the final version of the manuscript: JFL, AN, JAM, OO. Responsible for data integrity: JFL. Obtained funding: JFL. Guarantor: JFL had full access to all the data in the study and takes responsibility for the integrity of the data. JFL takes responsibility for the accuracy of the data analyses. All authors read and approved the final manuscript.

## Pre-publication history

The pre-publication history for this paper can be accessed here:

http://www.biomedcentral.com/1471-230X/13/89/prepub

## Supplementary Material

Additional file 1 Characteristics of study participants with diagnosis of CD <2 years of age. Click here for file
