# Supplementary material for: A large nationwide population-based case–control study of the association between intussusception and later celiac disease
Source: BMC Gastroenterol. 2013 May 16;13:89. doi: 10.1186/1471-230X-13-89 (PMC3661363; doi:10.1186/1471-230X-13-89)
Supplement: Additional file 1 — Characteristics of study participants with diagnosis of CD <2 years of age. [file 1471-230X-13-89-S1.doc]

***Characteristics of study participants with diagnosis of CD <2 years of age***

|  | ***Matched controls*** | ***Patients with celiac disease*** |
| --- | --- | --- |
| Total | 22,857 | 4,589 |
|  |  |  |
| Females (%) | 14,496 (63.4) | 2,913 (63.5) |
| Males (%) | 8,361 (36.6) | 1,676 (36.5) |
|  |  |  |
| *Calendar year* |  |  |
| -1989 | 5,813 (25.4) | 1,167 (25.4) |
| 1990-99 | 12,586 (55.1) | 2,525 (55.0) |
| 2000- | 4,458 (19.5) | 897 (19.5) |
|  |  |  |
| *Country of birth* |  |  |
| Nordic* | 22,734 (99.5) | 4,570 (99.6) |
| *Data on intussusception* | | |
| Intussusception (%) | 32 (0.14) | 6 (0.15) |
| Age at first intussusception, months* (median, range) | 6 (1-19) | 10 (4-15) |

* Sweden, Denmark, Finland, Norway and Iceland.
